# Comparing food literacy by grade, sex, and food education exposure: construct validation of the food literacy BITE scale

**DOI:** 10.3389/fnut.2026.1819437

**Published:** 2026-06-02

**Authors:** Christine St. Pierre, Jeffrey B. Bingenheimer, Rob M. van Dam, Jennifer M. Sacheck

**Affiliations:** 1 Department of Exercise and Nutrition Sciences, Milken Institute School of Public Health, The George Washington University, Washington, DC, United States; 2 Department of Prevention and Community Health, Milken Institute School of Public Health, The George Washington University, Washington, DC, United States; 3 Department of Behavioral and Social Sciences, School of Public Health, Brown University, Providence, RI, United States

**Keywords:** children, food education, food literacy, measurement properties, scale development

## Abstract

**Background:**

Food literacy provides a framework for understanding dietary behaviors in children and can inform priorities for and evaluate the effectiveness of food education interventions. The 4-dimension Food Literacy BITE scale is built on a child-centered food literacy framework and has been structurally validated in U.S. elementary schoolchildren. We conducted additional validity testing to assess the tool’s generalizability, ability to differentiate mean food literacy by grade, sex, and food education exposure, and the association of food literacy score with food group consumption frequencies.

**Materials and methods:**

We administered the Food Literacy BITE scale and food frequency questionnaires to *n* = 690 4*^th^* and 5*^th^*-grade students from 10 elementary schools in Washington, DC, and Kentucky (52.5% 4*^th^* grade, 55.8% 4+ years of food education, 52.8% girls). Stepwise series of multiple-group confirmatory factor analysis models assessed measurement invariance by grade, sex, and school-level food education history. We examined group differences in overall food literacy score and dimension scores using latent means and used multilevel modeling for the food education exposure analysis to account for clustering within schools. We used weighted regression with robust standard errors to investigate associations between food literacy scores and food group consumption frequencies, aggregated by school and grade (*n* = 20).

**Results:**

Item interpretation was consistent across groups, with full scalar invariance established by grade and partial scalar invariance by sex and food education history (4+years vs. new/none). Girls scored higher than boys in the “confidence in everyday food skills” dimension (*p* = 0.03). Mean food literacy was higher in the group from schools offering 4+ years of experiential food education (*p* = 0.03), driven by higher scores in the “valuing shared food experiences” (*p* = 0.02) and “food systems and nutrition knowledge” (*p* = 0.04) dimensions. In the multilevel models, only the difference in “valuing shared food experiences” remained significant.

**Conclusion:**

Results support generalizable use of the Food Literacy BITE scale in the U.S. and areas with similar food practices and environments. The tool’s ability to differentiate between food education exposure history suggests it may be useful in identifying best practices for food education interventions targeting late childhood.

## 1 Introduction

Eating patterns during childhood and adolescence have been associated with several diet-related chronic disease outcomes (1–3). For more than 20 years, studies have consistently found a decrease in diet quality in adolescence and early adulthood compared with childhood (4–8). Late childhood into early adolescence is therefore a critical window for supporting the development of healthy dietary behaviors, which can be protective against declining diet quality during the transition into adulthood and food independence (7). School-based food education approaches can be particularly important avenues for improving children’s eating patterns, as they offer universal reach when embedded into the school day. Increasingly, children’s food education incorporates experiential food activities, such as gardening and cooking, that are associated with greater willingness to try new foods and higher consumption of nutrient-dense foods (9–11). More research is needed, however, to understand how these programs may influence the formation of longer-term dietary habits as children grow older.

The construct of food literacy provides a helpful framework for understanding the dietary behaviors that shape food habits and patterns by accounting for the complex interrelationships between individual-level drivers of food decisions, as well as their interaction with social and environmental contexts (12). Accurate food literacy assessment can therefore provide useful data for informing priority areas for children’s food and nutrition education approaches and evaluating education impact. To this end, a growing number of tools have been developed to measure food literacy in children and adolescents, with four recent tools—Dallant et al., in France, INFOLIT in India, DCFLQ in the Netherlands, and Ponce-Carreon et al., in Peru—focused specifically on this critical window of late childhood and early adolescence (13–16).

The various food literacy frameworks underlying existing tools, however, were all developed in adult-centered contexts and do not account for the different developmental stages and limited food independence in childhood and adolescence (17, 18). To address this gap, we previously undertook a scoping review of the peer-reviewed literature and developed a food literacy framework centered on the child and adolescent context (19). We identified four dimensions: Purposeful Engagement with Food, Valuing Shared Food Experiences, Food Systems and Nutrition Knowledge, and Confidence in Everyday Food Skills, that were consistent across both conceptual and measurement studies of food literacy in children and adolescents. This framework was the basis for developing the 18-item Food Literacy BITE (Building Informed, Thoughtful, Empowered eaters) scale for a late childhood target population (∼9–12 years). The four-dimensional structure with food literacy as a second-order factor was validated through confirmatory factor analysis (manuscript under review^1^).

Psychometric testing of food literacy assessment tools for children and adolescents has largely been limited to establishing the structural validity and internal consistency of the measurement model. To our knowledge, no existing children’s food literacy tools report on measurement invariance, the measurement property that signals whether the construct has the same meaning across different groups (e.g., sex, geographic location, socioeconomic status, etc.) (20). Establishing measurement invariance provides evidence that a scale is measuring the same thing (food literacy) across different populations and is an important prerequisite for comparing any mean score differences between populations (14, 15). Measurement invariance also indicates that a scale is generalizable, and broader use could provide consistent and comparable data for understanding food literacy levels and informing food and nutrition education best practices.

Evidence for a scale’s validity can further be supported with construct validity testing, which includes both differentiation by known groups expected to differ in latent levels of the construct, and associations of scale scores with measures of related constructs (21). Several existing studies on children and adolescents have found evidence for differences in dietary behaviors and food literacy-related constructs by age, sex, and food education exposure (22–25), but only three food literacy tools for this population report on hypothesis testing for differentiation between subgroups of these variables (13, 15, 26). Testing of associations between children or adolescents’ food literacy scores and scores on comparator instruments, such as dietary intake (16, 27) or health literacy scales (27, 28), is also limited.

In the present study, we sought to address these gaps in data on measurement invariance and construct validity through further testing of the Food Literacy BITE scale. Our aims were to: (1) assess measurement invariance by grade, sex, and school-level history of food education, (2) test for differentiation in mean overall food literacy and mean dimension scores by grade, sex, and school-level history of food education, and (3) test for associations between group-level mean food literacy scores and mean fruit, vegetable, sugar-sweetened beverage (SSB), sugary snack, and salty snack consumption frequency.

## 2 Materials and methods

The Food Literacy BITE scale was developed and tested using Classical Test Theory (CTT) following the guidelines outlined by DeVellis and Thorpe (29), and the development and initial testing for structural validity and internal consistency are described elsewhere (see text footnote 1). Briefly, we developed items for the four dimensions described above based on 23 reflective indicators that were also identified in our scoping review. We established item and scale-level content validity through two rounds of expert panel review (*n* = 10). Elementary students (*n* = 43) in 4*^th^* and 5*^th^* grade (∼9–12 years) participated in group cognitive interviews to provide feedback on scale comprehensibility in the target population, and we then tested the scale in May and June 2025 with 690 elementary schoolchildren across 10 different schools, primarily in urban settings. The final measurement model with 18 items, four first-order factors, and a second-order food literacy factor was validated through confirmatory factor analysis (CFA), and acceptable internal consistency was established with composite reliability values.

For the current study, we first assessed measurement invariance between groups for each of the following categorical variables: grade (4*^th^* vs. 5*^th^*), sex (girls vs. boys), and school-level history of food education (first year or none vs. 4+ years), which would allow us to then meaningfully compare group mean differences (20). To assess construct validity, we conducted a series of known-group validity tests, hypothesizing *a priori* that mean food literacy scores would be higher in 5*^th^* graders, in girls, and in the group of schools with 4+ years of food education programming. We also assessed construct validity through a series of convergent validity hypothesis tests, expecting we would find positive associations between mean food literacy score and mean fruit and vegetable consumption frequency and inverse associations between mean food literacy score and mean SSB, sugary snack, and salty snack consumption frequency. Evaluation of measurement invariance and hypothesis testing for construct validity are reported here in accordance with the COnsensus-based Standards for the selection of health Measurement INstruments (COSMIN) Reporting Guideline version 2.0 (30).

### 2.1 Participant recruitment

Our sample included students from ten public elementary schools: nine in Washington, DC, and one in Kentucky. Six of the schools had been providing all students with experiential food education classes embedded in the school day for four or more years and were purposively recruited in coordination with our community partner that administers the food education programming. The remaining four schools either did not offer embedded food education or were in their first year of doing so and were purposively recruited to add greater diversity in the socioeconomic and ethnic backgrounds of students in the sample and to include a school outside the urbanized Washington, DC setting.

All 4*^th^* and 5*^th^* grade students at each school were invited to participate. Parents/caregivers were notified through both written and digital communications that researchers would administer anonymous food literacy and food consumption questionnaires in their child’s class and were given the opportunity to opt out of participating. We used a passive consent process to facilitate participation from students who may be at greatest risk of poor diet quality and who are often underrepresented when active, written parent consent is required (31). Students gave verbal assent to participate at the start of the research visit, and those who opted out were provided with an alternative activity. No personally identifying information was collected from participants. Recruitment and data collection procedures and all questionnaire content were approved by the George Washington University Institutional Review Board (NCR 235223) and relevant school district research review boards where required.

### 2.2 Data collection and measures

We worked with administrators and teachers at each school to schedule research visits during a relevant class (nine schools), or an afterschool program (one school). Students were either divided into small groups of 8–12 to rotate through a “survey station” (eight schools) or completed the questionnaires as a larger class with trained researchers stationed throughout the room (two schools).

#### 2.2.1 Food literacy

Food literacy was assessed using the novel, 18-item Food Literacy BITE scale. The validated scale includes five items each measuring Purposeful Engagement with Food and Food Systems and Nutrition Knowledge and four items each measuring Valuing Shared Food Experiences and Confidence in Everyday Food Skills. All items use a five-point Likert-type response scale using emojis to represent response options form Really Agree to Really Disagree. The full validated scale is available as Supplementary materials. After explaining the purpose of the questionnaire and directions, researchers read each item of the Food Literacy BITE scale aloud, giving students time to select their responses.

#### 2.2.2 Food group consumption frequency

The second questionnaire asked students about their consumption frequency of fruit, vegetables, SSBs, sugary snacks, and salty snacks and was modeled on the School Physical Activity and Nutrition (SPAN) questionnaire. SPAN was designed for the short-term nature of children’s recall ability and for group-level differentiation to inform surveillance and planning (32), which aligns well with our objectives in this study. For each section—fruits, vegetables, sugar sweetened beverages, sugary snacks, and salty snacks—participants selected the number of times they consumed foods from the respective group on the previous day (0, 1, 2, or 3 or more). Example items and photos were included for each food group to enhance student understanding. Fruits were divided into eight subgroups and vegetables into five subgroups to provide more examples to prompt student recall following the same groupings as the FLEX dietary screener, which was developed for and validated in a similar age group (33). The full questionnaire is available as Supplementary materials.

#### 2.2.3 Demographic variables

Students provided their grade 4*^th^* or 5*^th^* and sex at birth (female, male, or prefer not to answer) at the end of each respective questionnaire. The history of food education variable was determined at the school level. Six of the schools were in at least their 4*^th^* year of partnership with an experiential food education program that is embedded into the school day. Each classroom participates in an extended food education class with a gardening component, cooking component, and grade-level standards-aligned lesson approximately nine times over the course of each school year. The remaining four schools, in the “new/no food education” category, were either in their first year as a partner school with this food education program or according to school administrators, did not have an embedded experiential food education program.

### 2.3 Data analysis

The Food Literacy BITE scale item responses were scored according to the response scale (Really Agree = 5, …, Really Disagree = 1) and entered into a spreadsheet for analysis. After excluding completed scales with more than two missing responses (*n* = 4), each scale item had less than 2% missing data. Item 4 had the most missing data, which is most likely due to the formatting of the item near example pictures on the first page of the questionnaire. No other patterns in missing data were detected, indicating that the missing-at-random assumption is plausible. Multiple imputation by chained equations (R package mice) was used to treat missing data. Five datasets were imputed, and one was selected for the analysis using a random number generator. For the current study, item responses were re-scaled (Really Agree = 4, …, Really Disagree = 0) for ease of interpreting mean and standard deviation values.

For measurement invariance and known-groups validity testing, we conducted a series of multiple group confirmatory factor analyses (MGCFA), a form of structural equation modeling that tests whether a measurement instrument measures the same latent construct consistently across different groups. In this study, we ran analyses comparing 4*^th^* and 5*^th^* grade groups, schools with 4 + years of experiential food education programming and schools with new or no embedded programming, and boys and girls. Once measurement invariance was established, we extracted the latent means for the groups from the MGCFA models to test for group level differences. Each analysis is explained in greater detail below.

#### 2.3.1 Measurement invariance

We carried out MGCFA modeling using the weighted least squares mean and variance adjusted (WLSMV) estimation, which is considered the most robust method for ordinal data (R package lavaan). The series of models tested progressively more constrained levels of measurement invariance: configural (equivalence of the measurement model across both groups), metric [equivalence of item loadings on the factors (i.e., food literacy dimensions)], and scalar (equivalence of both item loadings and thresholds). We used the conventional acceptable cut-offs for model fit indices to establish configural invariance: Comparative Fit Index (CFI) > 0.95, Root Mean Square Error of Approximation (RMSEA) < 0.06, and Standardized Root Mean-square Residual (SRMR) < 0.08 (34). In nested model comparison, we used ΔCFI < 0.01, ΔRMSEA < 0.015, and ΔSRMR < 0.015 as the criteria for accepting each progressively more constrained model (35). At the metric invariance testing stage, we also examined the model modification indices and item loadings for evidence of item-level non-invariance. We used a backward approach to sequentially release loading constraints on items that had both modification indices indicating localized misfit (>10), and a between-group loading difference > 0.10 (20). We evaluated changes in overall model fit indices as described above to achieve a partially invariant model, then proceeded to scalar invariance testing.

#### 2.3.2 Known-groups validity

We conducted univariate analyses to obtain mean scores and standard deviations for overall food literacy and each of the four dimensions, for the entire sample as well as by group within each of the three categorical variables. Between-group differences within each category were visually inspected with box plots, and we used Levine’s test to assess homogeneity of variance between groups. We analyzed raw mean differences using two-sample *t-*tests and used Cohen’s *d* to compare standardized effect sizes across dimensions. We compared these results with latent mean differences and effect sizes extracted from the scalar invariance MGCFA models, relying on the MGCFA results as the stronger evidence for between-group differences, given that they account for measurement error and systematic biases at the item level (36). We considered mean differences significant with a *p*-value < 0.05 and effect sizes small for 0.2 < *d* < 0.5, medium for 0.5 < *d* < 0.8, and large for *d* > 0.8. Since food education exposure was a school-level variable, we used a multilevel model analysis (linear mixed effects models) to examine whether any significant difference in latent means persisted when accounting for nesting of students within schools.

#### 2.3.3 Convergent validity

The food consumption frequency questionnaire responses were entered into a spreadsheet for analysis, and subgroup frequencies were summed for fruits and vegetables, respectively, to obtain total frequency for each of these groups. To address the possibility of overreporting if students consumed mixed dishes with fruits or vegetables from multiple subgroups, we visually inspected the data for total consumption frequency of each food group using boxplots and histograms and used 97.5% winsorization to minimize both the influence of extreme values and distortion of the data (37, 38). A total of 14 fruit observations and 15 vegetable observations were affected by the winsorization.

We used weighted regression with HC3 robust standard errors to examine associations between mean food literacy score and mean food group consumption frequencies, aggregated by school and grade (*n* = 20), as our primary convergent validity analysis. We conducted sensitivity analyses with aggregation at the school level only (*n* = 10) and at the classroom level (*n* = 38). We standardized food literacy group means prior to running regression models to aid interpretation of coefficients and considered coefficients significant with a *p*-value < 0.05.

## 3 Results

A total of 690 responses to the Food Literacy BITE scale were included in the analysis. In the sample, 52.5% were in 4*^th^* grade, 55.8% were at schools with 4+ years of experiential food education, and self-reported sex at birth was 45.5% female and 40.7% male (Table 1). Racial and socioeconomic demographics were estimated using school-level data, weighted by student enrollment at each school.

**TABLE 1:** Food literacy BITE scale sample demographics, *n* = 690.

Variables	*n*	Percentage
Grade		
4^th^	362	52.5
5^th^	328	47.5
School history of food education		
4 or more consecutive years (6 schools)	385	55.8
First year or no program (4 schools)	305	44.2
Sex at birth		
Female	314	45.5
Male	281	40.7
Prefer not to answer	95	13.8
Race/ethnicity(school-level data weighted by enrollment)		
Asian	–	2.8
Black	–	54.5
Hispanic/Latino	–	11.9
White	–	23.0
Multiple races	–	7.4
Economically disadvantaged (school-level data, weighted by enrollment)		39.4

### 3.1 Measurement invariance

Scalar invariance was supported for all three categorical variables, providing evidence that food literacy has the same meaning for both groups in each variable and group mean comparisons are valid. Full measurement invariance was supported across grades (Table 2). Across school-level history of food education, two items were freed from the equal loadings constraint based on modification indices and factor loadings in the metric invariance model, and partial scalar invariance in MG-CFA testing with these two items freed was established based on the standard thresholds for the CFI, RMSEA, and SRMR global fit indices (Table 2). The analysis for measurement invariance by sex was conducted on the subset of self-reported male and female participants (*n* = 595, 52.8% girls). One item had no responses of Really Disagree among girls, so we collapsed the Really Disagree and Disagree categories across both sexes for this analysis. In the metric invariance model by sex, one item was freed from the equal loadings constraint, and the overall fit of the partial invariance scalar model was acceptable (Table 2).

**TABLE 2:** Measurement invariance multiple group confirmatory factor analyses (MGCFA) model fit indices.

Variables	X^2^ (*df*)	X^2^ *p*-value	CFI	RMSEA [CI]	SRMR
Grade					
Configural invariance	473.31 (260)	<0.001	0.952	0.049 [0.042–0.056]	0.063
Metric invariance	479.74 (277)	<0.001	0.954	0.046 [0.039–0.053]	0.069
Scalar invariance	536.80 (326)	<0.001	0.952	0.043 [0.037–0.050]	0.067
Food education					
Configural invariance	457.99 (260)	<0.001	0.955	0.047 [0.040–0.054]	0.062
Metric invariance	501.32 (277)	<0.001	0.949	0.049 [0.042–0.055]	0.072
Partial metric invariance (q2 freed)	481.76 (276)	<0.001	0.953	0.047 [0.040–0.053]	0.071
Partial metric invariance (q2 + q20 freed)	464.75 (275)	<0.001	0.957	0.045 [0.038–0.052]	0.068
Partial scalar invariance	532.9 (324)	<0.001	0.952	0.043 [0.037–0.050]	0.066
Sex					
Configural invariance	430.08 (260)	<0.001	0.949	0.047 [0.039–0.055]	0.067
Metric invariance	433.48 (277)	<0.001	0.953	0.044 [0.036–0.051]	0.072
Partial metric invariance (q10 freed)	416.22 (276)	<0.001	0.958	0.041 [0.033–0.049]	0.070
Partial scalar invariance	461.82 (324)	<0.001	0.959	0.038 [0.030–0.045]	0.068

### 3.2 Known-groups validity

There were no differences in mean scores for overall food literacy or any of the dimensions between 4*^th^* and 5*^th^*-grade students (Supplementary Table 1). Comparison of raw group means by sex indicated higher overall mean food literacy in girls, driven by higher mean scores in the “valuing shared food experiences” and “confidence in everyday food skills” dimensions by a small effect size. The latent mean analysis indicated a higher mean among girls only in the “confidence in everyday food skills” dimension (Table 3).

**TABLE 3:** Food literacy mean differences by sex.

Variables	Full sample *n* = 690 mean (SD)	Girls *n* = 314 mean (SD)	Boys *n* = 281 mean (SD)	Raw mean difference, boys−girls (*t-*test *p*-value)	Effect size (*d)* raw mean difference	Latent mean difference^a^ (*p*-value)	Effect size (*d)* latent mean difference
Overall food literacy (max possible = 72)	55.05 (9.21)	56.55 (7.73)	55.11 (9.02)	−1.44* (0.038)	0.17	−0.01 (0.894)	0.01
Purposeful engagement with food (max possible = 20)	14.16 (3.65)	14.34 (3.49)	14.36 (3.49)	0.02 (0.946)	<0.001	0.05 (0.354)	0.11
Valuing shared food experiences (max possible = 16)	12.31 (2.85)	12.88 (2.10)	12.26 (3.01)	−0.62^**^ (0.005)	0.24	−0.04 (0.543)	0.08
Food systems and nutrition knowledge (max possible = 20)	14.46 (3.51)	14.69 (3.30)	14.51 (3.49)	−0.18 (0.514)	0.05	0.01 (0.868)	0.03
Confidence in everyday food skills (max possible = 16)	14.12 (2.38)	14.64 (1.64)	13.97 (2.47)	−0.67^***^ (<0.001)	0.32	−0.17* (0.034)	0.31

*^a^*Girls, reference group for latent mean difference.

*Indicates *p* < 0.05,

**indicates *p* < 0.01,

***indicates *p* < 0.001.

In the group of schools with 4+ years of food education programming, mean overall food literacy score was higher compared with the group with new or no embedded food education, driven by higher mean scores in the “valuing shared food experiences” and “food systems and nutrition knowledge” dimensions. Effect sizes for differences were small, and these findings were consistent across both raw mean and latent mean analyses (Table 4). In multilevel models accounting for clustering at the school level, the difference between groups in latent score remained significant for the “valuing shared food experiences” dimension but not for overall food literacy or the “food systems and nutrition knowledge” dimension. Variance in scores attributable to differences between schools was 6% for overall food literacy and ranged from 4% to 7% for each of the dimensions, based on the Intraclass Correlation Coefficients obtained from the multilevel models (Table 4).

**TABLE 4:** Food literacy mean differences by school-level history of food education.

Variables	Full sample *n* = 690 mean (SD)	New/no food ed. *n* = 305 mean (SD)	4+ years food ed. *n* = 385 mean (SD)	Raw mean difference, 4+- new/no (*t-*test *p*-value)	Effect size (*d)* raw mean difference	Latent mean difference^a^ (*p*-value)	Effect size (d) latent mean difference	MLM estimated difference^a^, β (*p*-value)	Intraclass correlation coefficient
Overall food literacy (max possible = 72)	55.05 (9.21)	53.79 (10.20)	56.05 (8.21)	2.26^**^ (0.002)	0.25	0.09* (0.027)	0.18	0.07 (0.363)	0.059
Purposeful engagement with food (max possible = 20)	14.16 (3.65)	13.85 (4.05)	14.40 (3.27)	0.55 (0.053)	0.15	0.04 (0.423)	0.09	0.11 (0.273)	0.043
Valuing shared food experiences (max possible = 16)	12.31 (2.85)	11.86 (3.02)	12.68 (2.65)	0.82^***^ (<0.001)	0.29	0.13* (0.016)	0.29	0.21 (0.048)	0.042
Food systems and nutrition knowledge (max possible = 20)	14.46 (3.51)	14.01 (3.74)	14.82 (3.28)	0.81^**^ (0.003)	0.23	0.09* (0.044)	0.29	0.16 (0.171)	0.071
Confidence in everyday food skills (max possible = 16)	14.12 (2.38)	14.07 (2.52)	14.15 (2.27)	0.08 (0.684)	0.03	−0.14 (0.057)	0.27	−0.04 (0.678)	0.035

*^a^*New/no food education, reference group.

*Indicates *p* < 0.05,

**indicates *p* < 0.01,

***indicates *p* < 0.001.

**FIGURE 1:**
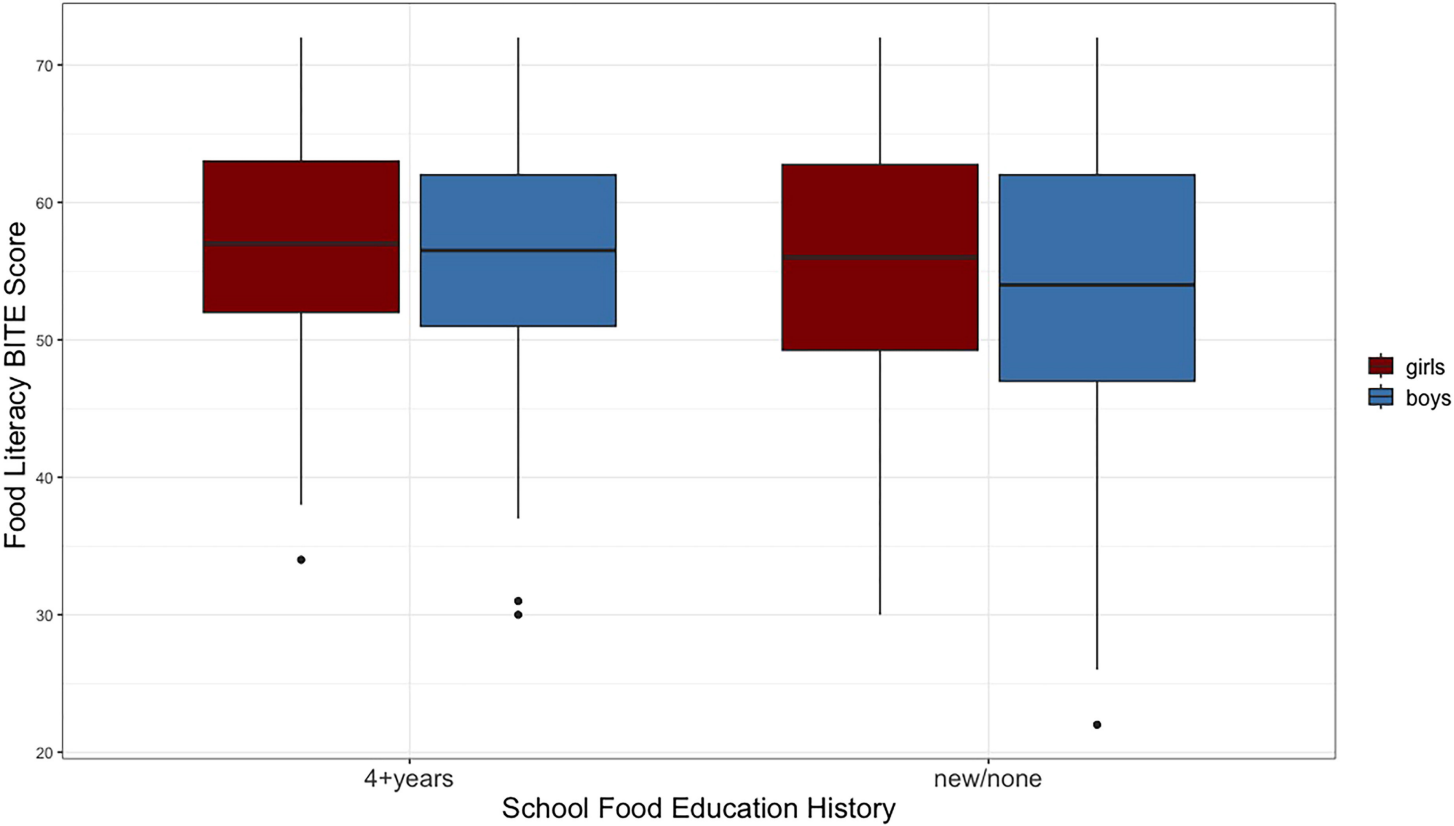
Food literacy score distribution by school food education history and sex.

After finding differences in mean food literacy scores by food education and by sex, we conducted a *post hoc* interaction analysis using a linear mixed-effects model to account for clustering within schools. The interaction term was non-significant (*p* = 0.302), indicating that the effect of food education exposure does not differ by sex (Figure 1).

### 3.3 Convergent validity

Data from a total of 680 food consumption frequency questionnaires were included in the convergent validity analyses, and all had complete responses for fruit consumption frequency. The number of missing responses was 4 for vegetables, 9 for SSBs, 10 for sugary snacks, and 8 for salty snacks. For each regression model, the weighted value for each school x grade grouping was set as the lower of the number of Food Literacy BITE responses or the number of responses for that particular food group (Supplementary Table 2). Weighted regression analyses of questionnaire responses aggregated at the school and grade level (*n* = 20) did not indicate statistically significant associations between mean food literacy scores and mean consumption frequency for any of the food groups, although the coefficient and correlation signs were in the expected direction for each food group (Table 5). Sensitivity analyses by school (*n* = 10) and by classroom (*n* = 38) showed similar results.

**TABLE 5:** Weighted regression estimates of association between mean food literacy score and mean food group consumption frequency.

Outcome: mean consumption frequency	Predictor: standardized mean food literacy score			
	β	SE^a^	*P*-value	Weighted Pearson correlation, *r*
Model 1: fruit	0.21	0.26	0.451	0.18
Model 2: vegetables	0.06	0.13	0.627	0.12
Model 3: SSBs	−0.10	0.08	0.320	−0.24
Model 4: sugary snacks	−0.07	0.06	0.252	−0.27
Model 5: salty snacks	−0.07	0.07	0.286	−0.25

*^a^*HC3 standard error estimate.

## 4 Discussion

The Food Literacy BITE scale is to our knowledge, the first food literacy assessment tool for children or adolescents developed from a conceptual framework explicitly centered on this population that accounts for their developing autonomy and critical analysis skills. Although the scale’s structural model was previously verified, the current study adds to the knowledge of its measurement properties in several important ways. Our findings (1) support generalizable use of the Food Literacy BITE scale in late childhood populations beyond the initial psychometric testing locations, (2) suggest it can discriminate between differing food literacy levels by food education history, and (3) highlight next steps for refining and improving the tool.

Our findings of scalar-level measurement invariance by grade, food education history, and sex in the current study provide evidence that the Food Literacy BITE scale measures the same latent construct across different groups within the sample population—indicating suitability for more generalizable use and valid subsequent comparison of food literacy levels between groups. Food literacy tools are often context-specific (13, 14, 17); a natural outflow of the context-specific nature of dietary practices and food environments (39, 40). Identifying food education approaches that have the greatest effect on children’s dietary behaviors, however, requires consistency and comparability in outcome measures (41, 42). The Food Literacy BITE scale could thus be a useful tool for comparing the effects of various food education strategies and informing new interventions in the U.S. and similar Western countries.

As hypothesized, we found higher mean food literacy scores in girls compared with boys and in the group of schools with 4+ years of regular experiential food education embedded into the school day compared to schools with new or no universal programming; however, we did not find a difference in mean scores by grade. These findings are consistent with previous studies comparing raw mean food literacy scores by sex and school-based food education exposure in late childhood in France (13) and early adolescence in China (26). The lack of difference by grade in our study suggests that one year is not sufficient time to observe a meaningful differentiation in food literacy levels.

The current study extended known-groups validity analyses to include latent mean comparisons, ensuring we were controlling for measurement error and other systematic biases that may affect raw mean comparisons. The difference in mean food literacy between girls and boys disappeared in the latent mean analysis, indicating that factors such as systematic response style differences by sex or potential confounders such as socioeconomic status and cultural background may be contributing to the observed difference in raw means. Mean differences by school-level food education history were consistent across both raw and latent mean analyses, but after accounting for nesting of students within schools with multilevel modeling, only the difference in ‘valuing shared food experiences’ mean score remained significant. We were highly specific about our food education variable, verifying that the “exposure” schools were in at least their 4^th^ consecutive year of offering universal food education with hands-on cooking and gardening experiences. Our persistent dimension-specific findings suggest that this food education exposure may be particularly effective in helping students build the social infrastructure that supports healthy food choices (43, 44).

During our initial structural validation analysis of the Food Literacy BITE scale, we noted that the “confidence in everyday food skills dimension” had the most highly skewed response distribution, and that item revision could improve differentiation across individuals’ varying latent levels of the dimension (19). The current study reinforces these findings, with this dimension showing the greatest variability between raw and latent mean analyses. Deeper item-level analyses can help guide can help guide future revisions, but MGCFA global fit indices and item loadings nevertheless support the acceptability of using the Food Literacy BITE scale in its current form.

In our convergent validity analyses, associations between group-level mean food literacy scores and mean food group consumption frequencies were in the expected direction, but not statistically significant, as group level aggregation resulted in power to detect only moderate to large associations (>0.55). The strength of association between food literacy and consumption frequency in our analysis was small to moderate for each of the food groups—in line with findings from the two previous children’s food literacy scale validation studies that conducted similar testing (16, 27). Future, more highly powered studies with individual-level comparisons could provide greater clarity regarding associations and also explore potential differences in the magnitude of each food literacy dimension’s relationship with food consumption.

This study sets several precedents for expanding psychometric testing of food literacy assessment tools for children and adolescents. Measurement invariance should be established prior to group mean comparisons and can serve as an indicator for the extent of a tool’s generalizability. Known-groups validity testing using latent means and multilevel modeling where appropriate can help ensure that observed group mean differences are truly a result of different levels of the latent construct rather than measurement artifacts. Similarly, convergent validity assessment methods that account for potential clustering (e.g., within schools) can better discern true associations between scale scores and comparator instruments.

This study also has several limitations. Data from the same sample were used in the structural validity CFA analyses and the MGCFA analyses in this study. While this is common practice for novel questionnaire development, replication of the findings in an independent sample would strengthen the robustness of the scale validation. While the inclusion of two geographic locations in our testing sample strengthens the argument for generalizability use of the Food Literacy BITE scale in the U.S., its international generalizability may be limited by sharper between-country differences in cultural practices, food environments, and socioeconomic variables. In convergent validity analysis, group-level data aggregation reduced our power to detect associations between food literacy and food group consumption frequency. Finally, as with any measurement scale, validation is an ongoing process, and construct validity testing should continue with any future use of this tool.

## 5 Conclusion

The Food Literacy BITE scale addresses important gaps in measuring food literacy from a child-centered framework, and robust psychometric testing supports the measurement model, the scale’s consistency in measuring the same latent construct, and its ability to differentiate between different levels of the latent construct across known groups. Furthermore, this study’s findings suggest that universal, school-based experiential food education programs are an effective strategy for building children’s food literacy and thus supporting them in the development of healthy dietary habits. Future use of the Food Literacy BITE scale can provide important data to better understand the relationships between food education, food literacy, and dietary habit formation during the critical late-childhood developmental window and inform effective interventions to mitigate diet quality declines in adolescence and their subsCequent adverse health effects.

## Data Availability

The raw data supporting the conclusions of this article will be made available by the authors, without undue reservation.

## Footnotes

1 St. Pierre C, Bingenheimer JB, van Dam RM, Sacheck JM. *The Food Literacy BITE (Building Informed, Thoughtful, Empowered Eaters) Scale: Development and measurement properties in U.S. elementary schoolchildren* (under review).
