# Supplementary material for: Comparing food literacy by grade, sex, and food education exposure: construct validation of the food literacy BITE scale
Source: Front Nutr. 2026 Jun 2;13:1819437. doi: 10.3389/fnut.2026.1819437 (PMC13268878; doi:10.3389/fnut.2026.1819437)
Supplement: Supplementary file 1 [file Data_Sheet_1.pdf]

Food Literacy BITE Scale

For each statement, please circle the emoji that best describes you.
There are no right or wrong answers!

	Really Agree	Agree	I'm not sure	Disagree	Really Disagree
1. I enjoy eating many different fruits.	😍	😊	😐	😕	😫
2. I like to try fruits that are new to me or prepared in a new way.	😍	😊	😐	😕	😫
3. I enjoy eating many different vegetables.	😍	😊	😐	😕	😫
4. I like to try vegetables that are new to me or prepared in a new way.	😍	😊	😐	😕	😫
5. I like to try foods from different places around the world.	😍	😊	😐	😕	😫

<u>Examples of fruits</u>

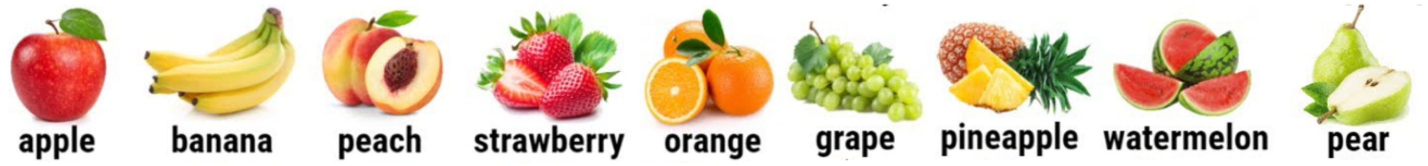

<u>Examples of vegetables</u>

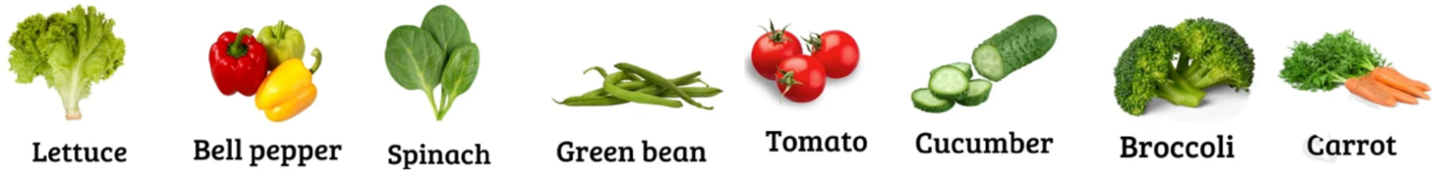

	Really Agree	Agree	I’m not sure	Disagree	Really Disagree
6. I like eating together with my family or friends.	😍	😊	😐	😕	😫
7. I like to cook with my family or friends.	😍	😊	😐	😕	😫
8. I like to share things I learn about food and nutrition with my family.	😍	😊	😐	😕	😫
9. I often talk with my friends about the foods we like to eat.	😍	😊	😐	😕	😫
10. I can name the things that fruit or vegetable plants need to grow.	😍	😊	😐	😕	😫
11. I can name some fruits and vegetables that grow in the part of the country where I live.	😍	😊	😐	😕	😫
12. I can explain the steps for how apples get from farms to my home.	😍	😊	😐	😕	😫
13. I can find the list of ingredients on a box of cereal.	😍	😊	😐	😕	😫

	Really Agree	Agree	I’m not sure	Disagree	Really Disagree
14. I can give some examples of foods that help keep my body healthy and strong.					
15. I know how to use kitchen tools like measuring cups, peelers, and graters.					
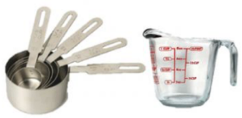 Measuring cups 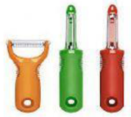 Peelers 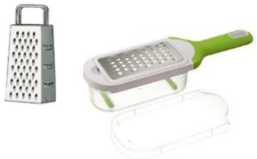 Graters					
16. I can follow a recipe to make food.					
17. I know how to use a knife to cut fruits or vegetables on my own or with help from an adult.					
18. I can prepare some foods for myself like a sandwich or salad.					

Please circle your grade: 4th 5th

Please circle your sex: Female Male Prefer not to answer
