# Supplementary material for: Comparing food literacy by grade, sex, and food education exposure: construct validation of the food literacy BITE scale
Source: Front Nutr. 2026 Jun 2;13:1819437. doi: 10.3389/fnut.2026.1819437 (PMC13268878; doi:10.3389/fnut.2026.1819437)
Supplement: Supplementary file 2 [file Data_Sheet_2.pdf]

THE GEORGE WASHINGTON UNIVERSITY
WASHINGTON, DC

Food Group Consumption Frequency Questionnaire

1. Fruits

Think about all the times you ate yesterday, from when you got up, until you went to bed. Think about **all** the fruit you ate **yesterday,** including fresh, frozen, canned and dried. ***Do not count juices.*** Read each row and choose how many times you ate fruit from that group **yesterday.**

Type of Fruit	Examples	How many times did you eat these fruits yesterday?			
		0 times	1 time	2 times	3 or more times
Apples and pears	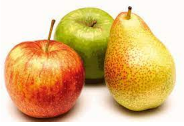	☐	☐	☐	☐
Citrus fruits (such as oranges, tangerines, clementines, and grapefruits)	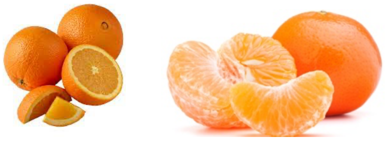	☐	☐	☐	☐
Grapes and Berries (such as blackberries, cranberries, blueberries, raspberries and strawberries)	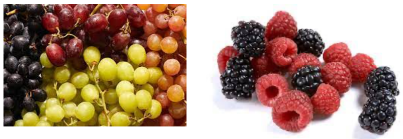	☐	☐	☐	☐
Melons (such as cantaloupe, honeydew and watermelon)	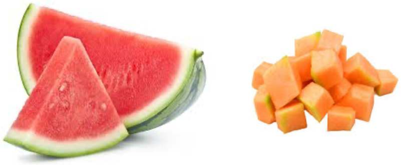	☐	☐	☐	☐
Fruit with pits (such as peaches, plums, nectarines, cherries, and apricots)	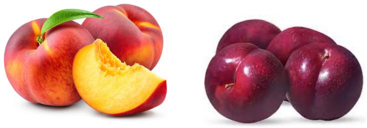	☐	☐	☐	☐
Tropical fruit (such as bananas, pineapple, kiwi, mangoes, papayas, pomegranates, guavas and coconut)	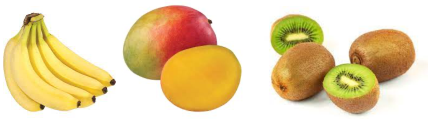	☐	☐	☐	☐
Dried fruit such as raisins, Craisins, dried apples, dried pineapple, and other dried fruit	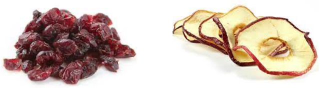	☐	☐	☐	☐
Packaged fruit (such as fruit cups, fruit cocktail, applesauce, fruit pouches like Gogo Squeez)	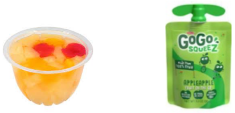	☐	☐	☐	☐

THE GEORGE WASHINGTON UNIVERSITY
WASHINGTON, DC

2. Vegetables

Think about all the times you ate yesterday, from when you got up, until you went to bed. Now think about **all** the vegetables you ate **yesterday,** including raw or cooked and fresh, frozen, or canned. Read each row and choose how many times you ate vegetables from that group **yesterday.** Don't forget to include vegetables in mixed dishes like chili or pasta!

Type of vegetable	Examples	How many times did you eat these vegetables yesterday?			
		None	1 time	2 times	3 or more times
Salad and dark green vegetables (such as lettuce, spinach, kale, broccoli, collard greens and bok choy)	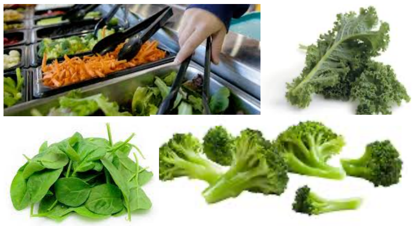	☐	☐	☐	☐
Starchy vegetables (such as corn, green peas, potatoes, plantains, and cassava. **Do not count fried potatoes or French fries.**	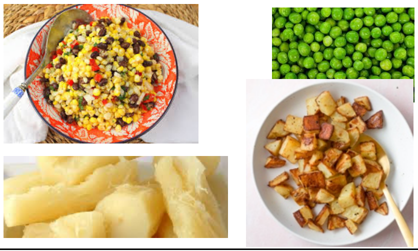	☐	☐	☐	☐
Red and orange vegetables (such as carrots, tomatoes, bell peppers, sweet potatoes, butternut squash, and pumpkin)	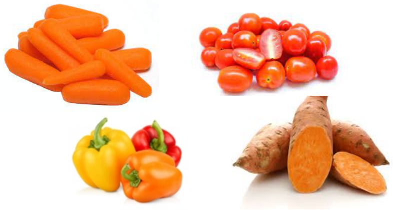	☐	☐	☐	☐
Beans (such as black beans, black-eyed peas, chickpeas, kidney beans, lima beans, white beans, pinto beans, refried beans, and soybeans/edamame)	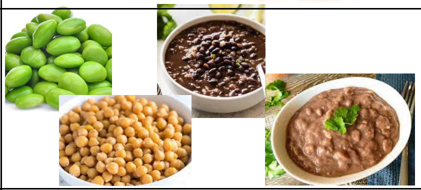	☐	☐	☐	☐
Other vegetables (such as cucumber, celery, zucchini, green beans, cauliflower, avocado, cabbage, mushrooms, eggplant, brussels sprouts, turnips, okra)	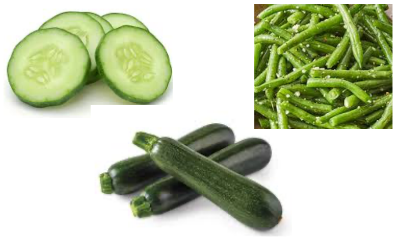	☐	☐	☐	☐

THE GEORGE WASHINGTON UNIVERSITY
WASHINGTON, DC

3. Other Foods/Drinks

Think about all the times you ate or drank something yesterday, from when you got up, until you went to bed. Read each row and choose how many times you ate or drank a food from that group **yesterday.**!

Food/Drink Groups	Examples	How many times did you eat or drink something from this group yesterday?			
		None	1 time	2 times	3 or more times
**100%** fruit juice (like apple juice, orange juice, grape juice)	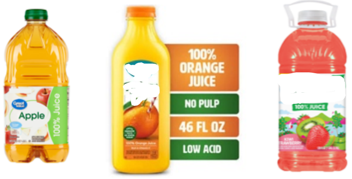	☐	☐	☐	☐
Drinks with sugar added, like regular soda, punch, sports drinks or fruit flavored drinks	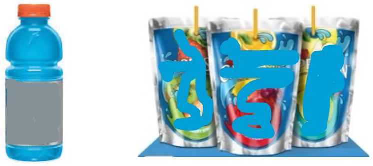	☐	☐	☐	☐
Cookies, brownies, pies, cake, cupcakes, donuts, sweet rolls, frozen desserts (ice cream or popsicles), or candy	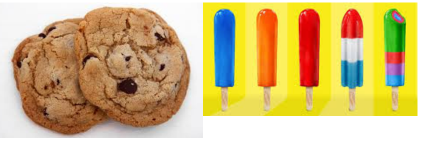	☐	☐	☐	☐
Chips (like potato chips, tortilla chips, hot fries), or French fries	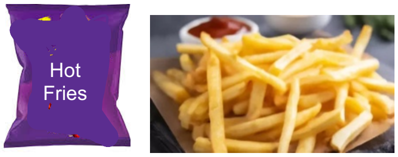	☐	☐	☐	☐

Please circle your grade: 4th 5th

Please circle your sex: Female Male Prefer not to answer
