# Supplementary material for: Comparing food literacy by grade, sex, and food education exposure: construct validation of the food literacy BITE scale
Source: Front Nutr. 2026 Jun 2;13:1819437. doi: 10.3389/fnut.2026.1819437 (PMC13268878; doi:10.3389/fnut.2026.1819437)
Supplement: Supplementary file 3 [file Table_1.docx]

**Table S1. Food Literacy Mean Differences by Grade**

|  | **Full Sample**  **n = 690**  **mean (SD)** | **4^th^ grade**  **n = 362**  **mean (SD)** | **5^th^ grade**  **n = 328**  **mean (SD)** | **Raw mean difference,**  **5^th^ - 4^th^**  **(*t-*test**  **p-value)** | **Effect size (*d)* raw mean difference** | **Latent mean difference^a^**  **(p-value)** | **Effect size (*d)* latent mean difference** |
| --- | --- | --- | --- | --- | --- | --- | --- |
| Overall Food Literacy  (max possible = 72) | 55.05  (9.21) | 54.79  (9.50) | 55.33  (8.89) | 0.54  (0.441) | 0.06 | 0.01  (0.861) | 0.01 |
| Purposeful Engagement with Food (max possible = 20) | 14.16  (3.65) | 14.02  (3.75) | 14.31  (3.53) | 0.29  (0.293) | 0.08 | ~0.00  (0.931) | 0.01 |
| Valuing Shared Food Experiences  (max possible = 16) | 12.31  (2.85) | 12.31  (2.90) | 12.31  (2.83) | 0.00  (0.993) | <0.001 | -0.03  (0.527) | 0.08 |
| Food Systems and Nutrition Knowledge  (max possible = 20) | 14.46  (3.51) | 14.42  (3.65) | 14.50  (3.34) | 0.08  (0.797) | 0.02 | -0.05  (0.246) | 0.17 |
| Confidence in Everyday Food Skills  (max possible = 16) | 14.12  (2.38) | 14.03  (2.31) | 14.21  (2.48) | 0.18  (0.332) | 0.07 | 0.12  (0.099) | 0.23 |

^a^4^th^ grade = reference group for latent mean difference
