# Supplementary material for: Comparing food literacy by grade, sex, and food education exposure: construct validation of the food literacy BITE scale
Source: Front Nutr. 2026 Jun 2;13:1819437. doi: 10.3389/fnut.2026.1819437 (PMC13268878; doi:10.3389/fnut.2026.1819437)
Supplement: Supplementary file 4 [file Table_2.docx]

**Table S2. Food Literacy and Food Consumption Frequency Responses by School and Grade**

|  |  | **Food literacy *n*** | **Fruit *n*** | **Vegetable *n*** | **SSB *n*** | **Sugary Snacks *n*** | **Salty Snacks *n*** |
| --- | --- | --- | --- | --- | --- | --- | --- |
| School 1 | 4^th^ | 42 | 41 | 40 | 41 | 41 | 41 |
|  | 5^th^ | 24 | 24 | 24 | 24 | 24 | 24 |
| School 2 | 4^th^ | 37 | 37 | 37 | 36 | 36 | 37 |
|  | 5^th^ | 25 | 25 | 25 | 25 | 25 | 25 |
| School 3 | 4^th^ | 23 | 23 | 23 | 23 | 22 | 22 |
|  | 5^th^ | 33 | 31 | 31 | 31 | 30 | 31 |
| School 4 | 4^th^ | 32 | 33 | 33 | 33 | 33 | 33 |
|  | 5^th^ | 42 | 43 | 43 | 43 | 43 | 42 |
| School 5 | 4^th^ | 37 | 35 | 35 | 35 | 35 | 35 |
|  | 5^th^ | 38 | 36 | 36 | 36 | 36 | 36 |
| School 6 | 4^th^ | 28 | 28 | 28 | 28 | 28 | 28 |
|  | 5^th^ | 24 | 23 | 22 | 22 | 22 | 22 |
| School 7 | 4^th^ | 49 | 50 | 48 | 48 | 49 | 49 |
|  | 5^th^ | 49 | 47 | 47 | 47 | 46 | 47 |
| School 8 | 4^th^ | 67 | 65 | 65 | 61 | 63 | 63 |
|  | 5^th^ | 50 | 50 | 50 | 50 | 50 | 50 |
| School 9 | 4^th^ | 37 | 37 | 37 | 36 | 36 | 36 |
|  | 5^th^ | 37 | 36 | 36 | 36 | 36 | 36 |
| School 10 | 4^th^ | 10 | 10 | 10 | 10 | 9 | 10 |
|  | 5^th^ | 6 | 6 | 6 | 6 | 6 | 5 |
